# Integrating the Hospital Frailty Risk Score into Explainable Machine Learning to Predict Mortality in Older Adults with Pneumonia: A Chilean Population-Based Study

**DOI:** 10.3390/diagnostics16101506

**Published:** 2026-05-15

**Authors:** Yeny Concha-Cisternas, Eduardo Guzmán-Muñoz, Manuel Vásquez-Muñoz, Claudia Troncoso-Pantoja, Lincoyán Fernández-Huerta, Rodrigo Olivares, Exal Garcia-Carrillo, Iván Molina-Marquez, Jorge Leschot Gatica, Rodrigo Yañez-Sepúlveda

**Affiliations:** 1 Escuela de Kinesiología, Facultad de Salud, Universidad Santo Tomás, Talca 3460000, Chile; 2 Vicerrectoría de Investigación e Innovación, Universidad Arturo Prat, Iquique 1100000, Chile; 3 Escuela de Fonoaudiología, Facultad de Ciencias de la Salud, Universidad Autónoma de Chile, Talca 3460000, Chile; 4 Center for Health Data Observation and Analysis (CADS), School of Medicine and Health Sciences, Universidad Mayor, Santiago 8580745, Chile; manuel.vasquez@umayor.cl; 5 Escuela de Medicina, Facultad de Medicina y Ciencias de la Salud, Universidad Mayor, Santiago 8580745, Chile; 6 Centro de Investigación en Educación y Desarrollo (CIEDE-UCSC), Departamento de Salud Pública, Facultad de Medicina, Universidad Católica de la Santísima Concepción, Concepción 4030000, Chile; ctroncosop@ucsc.cl; 7 Escuela de Kinesiología, Facultad de Ciencias de la Rehabilitación y Calidad de Vida, Universidad San Sebastián, Concepción 4030000, Chile; lincoyan.fernandez@uss.cl; 8 Escuela de Ingeniería Informática, Universidad de Valparaíso, Valparaíso 2340000, Chile; rodrigo.olivares@uv.cl; 9 Department of Physical Activity Sciences, Faculty of Education Sciences, Universidad Católica del Maule, Talca 3480112, Chile; exal.garcia@gmail.com; 10 Department of Physical Activity Sciences, Universidad de Los Lagos, Osorno 5290000, Chile; 11 Pedagogía en Educación Física, Facultad de Educación, Universidad Adventista de Chile, Chillán 3780000, Chile; ivanmolina@unach.cl; 12 Programa de Doctorado en Ciencias de la Actividad Física, Universidad Católica del Maule, Talca 3460000, Chile; 13 Escuela de Ciencias de la Actividad Física, Facultad de Salud y Ciencias Sociales, Universidad de las Américas, Concepción 4030000, Chile; jleschotg@gmail.com; 14 Facultad de Educación y Humanidades, Escuela de Ciencias del Deporte, Universidad Andres Bello, Viña del Mar 2200055, Chile; rodrigo.yanez.s@unab.cl; 15 School of Medicine, Universidad Espíritu Santo, Samborondón 092301, Ecuador

**Keywords:** community-acquired pneumonia, machine learning, frailty, hospital frailty risk score, mortality prediction

## Abstract

**Background/Objectives**: Community-acquired pneumonia (CAP) is a leading cause of mortality in older adults. Traditional prognostic scores may underestimate risk in frail patients by assuming linear relationships between predictors and outcomes. This study aimed to develop and validate explainable machine learning models integrating the administrative Hospital Frailty Risk Score (HFRS) to predict in-hospital mortality in a nationwide cohort of older adults in Chile. **Methods**: A retrospective cohort study was conducted using anonymized hospital discharge records from the Chilean National Health Fund (FONASA), including 58,306 hospitalization episodes of adults aged ≥60 years across 72 public hospitals. Fourteen supervised machine learning algorithms were trained using five routinely collected predictors: age, sex, HFRS, Charlson Comorbidity Index, and length of stay. Model performance was evaluated on an independent test set using AUC-ROC. SHAP (SHapley Additive exPlanations) values were calculated to assess global and individual predictor contributions. **Results**: The Extra Trees classifier achieved the highest discriminative performance (AUC-ROC 0.862), outperforming logistic regression (0.642) and other linear models. SHAP analyses identified HFRS as the most influential predictor (mean |SHAP| = 0.66), followed by length of stay, age, and comorbidities. **Conclusions**: Ensemble tree-based models incorporating administrative frailty measures provide superior mortality prediction compared to traditional linear approaches. Frailty emerged as the primary driver of risk, supporting scalable early stratification using routinely available hospital data.

## 1. Introduction

Community-acquired pneumonia (CAP) represents one of the leading causes of morbidity and mortality worldwide and remains among the most lethal infectious diseases in older adults [1,2]. According to data from the Global Burden of Disease (GBD) study, lower respiratory tract infections accounted for approximately 2.5 million deaths in 2019, with the highest mortality rates observed in individuals aged ≥70 years. This increased vulnerability is largely attributable to immunosenescence and the progressive decline in pulmonary physiological reserve associated with aging [3,4]. In the Latin American context—and particularly in Chile—this scenario is especially concerning due to the rapid demographic shift toward population aging. National epidemiological data indicate that the incidence of CAP in Chilean older adults exceeds 15 cases per 1000 person-years [5], and in-hospital mortality reaches 19.3% [6]. This substantial disease burden imposes significant pressure on the public healthcare system and markedly increases hospitalization costs, particularly among patients requiring admission to intensive care units (ICUs), where resource utilization and expenditures are considerably higher [7].

Prognostic assessment in older adults hospitalized with CAP challenges traditional paradigms. Although advanced age and multimorbidity are well-established risk factors [6,8,9], emerging evidence suggests that frailty—defined as a state of cumulative biological vulnerability to acute stressors—constitutes a more accurate predictor of mortality than the arithmetic sum of comorbidities or chronological age alone [6,10]. In this context, and given the need to systematically assess frailty among hospitalized patients with CAP, the Hospital Frailty Risk Score (HFRS) has been validated as an administrative data-derived tool that provides an objective, standardized, and cost-effective approach to identifying individuals at high risk of adverse outcomes [11,12]. Early identification of this high-risk phenotype is critical, as frailty is strongly associated with accelerated clinical deterioration and a substantially increased likelihood of admission to Intensive Care Units [13,14]. Within this framework, the timely initiation of advanced life-support measures—including invasive mechanical ventilation and hemodynamic stabilization—alongside intensified antimicrobial therapy, is pivotal for modifying disease trajectory and improving survival [13]. Effective risk stratification not only enhances clinical outcomes but also optimizes the allocation of critical care resources and mitigates the financial burden imposed on healthcare systems [13,15].

To guide risk stratification in CAP, prognostic tools such as CURB-65 and the Pneumonia Severity Index (PSI) have historically been employed, facilitating standardized clinical decision-making [16,17,18]; however, these instruments present important structural limitations, as they fail to fully capture the complex interactions among clinical variables when predicting mortality, largely due to their assumption of linear relationships between predictors and outcomes [19]. This methodological rigidity may lead to underestimation of risk in frail older adults with multimorbidity who do not present with overt hemodynamic instability, thereby limiting their ability to accurately anticipate the need for intensive care [20,21].

In this context, machine learning offers substantial potential for large-scale data processing and improved predictive accuracy [13,20,22]. Machine learning enables computational systems to identify complex patterns and relationships within data without explicit programming, encompassing supervised, unsupervised, and reinforcement learning approaches [23]. Its adoption has expanded considerably in clinical medicine, particularly in critical care settings, where it assists clinicians in managing high-dimensional information more efficiently [24,25]. While earlier studies relied on single-model approaches—often resulting in suboptimal predictive performance [26]—tree-based ensemble methods such as Random Forest, Gradient Boosting, and LightGBM have demonstrated superior capacity to model nonlinear interactions within heterogeneous clinical datasets [13]. Recent investigations have reported that these models achieve robust area under the curve (AUC) values, consistently exceeding the 0.80 threshold and even reaching 0.84–0.85 in external validation cohorts, representing a substantial improvement over conventional logistic regression and traditional severity scoring systems [13]. However, previous studies have differed substantially in their clinical settings, data sources, and predictor structures, including intensive care populations, single-center cohorts, and models incorporating clinical or laboratory variables, which limits direct comparability across studies and makes it difficult to determine whether similar performance can be achieved using routinely collected administrative data alone [13,20,22].

Finally, the integration of explainable artificial intelligence techniques, such as SHapley Additive exPlanations (SHAP), helps overcome the “black box” limitation by providing analytical transparency that facilitates clinical interpretation of how factors such as frailty drive individual risk [27,28]. This approach offers greater interpretability than traditional scoring systems and other machine learning methods, enabling clinically meaningful understanding of complex models without compromising predictive accuracy [27,28].

Despite these advances, validation of such models using nationwide administrative data in Latin America remains limited. Moreover, the specific contribution of an administrative frailty measure such as the Hospital Frailty Risk Score within explainable machine learning models for mortality prediction in older adults with CAP has not been sufficiently examined at a national level. Therefore, the present study aimed to develop and internally validate supervised machine learning models to predict in-hospital mortality in a national cohort of older adults with CAP in Chile. We hypothesized that integrating HFRS into tree-based algorithms would yield superior discriminative performance compared with linear models, enabling more robust, personalized, and clinically interpretable risk stratification.

In this context, the major contributions of the present study are threefold. First, this study develops and internally validates supervised machine learning models for predicting in-hospital mortality among older adults hospitalized with community-acquired pneumonia using a large nationwide administrative database from the Chilean public healthcare system. Second, it incorporates the Hospital Frailty Risk Score as a routinely available administrative measure of frailty, allowing its relative contribution to mortality prediction to be quantified alongside age, sex, comorbidity burden, and length of hospital stay. Third, by comparing 14 supervised learning algorithms and applying SHAP-based explainability methods to the best-performing model, this study provides both predictive and interpretable evidence supporting the feasibility of frailty-informed risk stratification using routinely collected hospital data in a Latin American setting.

## 2. Materials and Methods

This retrospective cohort study developed and internally validated supervised machine learning models to predict in-hospital mortality among older adults hospitalized with pneumonia in Chile. The study relied on anonymized hospital discharge records from the Chilean National Health Fund (Fondo Nacional de Salud, FONASA), which maintains a standardized administrative database integrating information from 72 public hospitals across the national healthcare system that provide moderate- to high-complexity care. The registry contains routinely collected demographic, diagnostic, clinical risk, and discharge outcome information for each hospitalization episode. All stages of model development and reporting adhered to the TRIPOD-AI recommendations to ensure methodological transparency, reproducibility, and clinical interpretability [29].

### 2.1. Participants

All hospital admissions involving adults aged 60 years or older with a principal diagnosis of CAP between 1 January 2019 and 31 December 2024 were considered eligible for inclusion. Cases were identified according to International Classification of Diseases, Tenth Revision (ICD-10) codes J12–J18, which include viral, bacterial, and unspecified forms of pneumonia [30]. Pneumonia episodes related to COVID-19 were excluded using SARS-CoV-2-specific codes in order to minimize etiological heterogeneity. However, because testing practices were not systematically recorded in the administrative registry, complete clinical exclusion of COVID-19 could not be independently confirmed. To preserve the representativeness of this nationwide cohort, exclusions were intentionally restricted to data quality issues only, including missing information on age, sex, discharge status, or absence of all predictor variables. Encrypted admission and discharge identifiers were cross-checked to ensure internal consistency and eliminate duplicate or transfer records, with each observation corresponding to a unique hospitalization episode. Clinical variables not routinely captured in administrative datasets—such as laboratory parameters, physiological measurements, or imaging confirmation—were unavailable but not used as exclusion criteria.

After applying these procedures, the final analytic cohort comprised 58,306 hospitalization episodes of older adults treated within the Chilean public hospital network.

### 2.2. Outcomes

The primary outcome was in-hospital mortality, defined as death occurring during the same hospitalization episode for pneumonia. Mortality status was extracted from the standardized discharge condition field within the FONASA administrative registry, which systematically records each patient’s vital status at the time of hospital discharge.

In-hospital mortality (death) was automatically identified through string matching of standardized discharge status labels (e.g., the Spanish term “fallecido”) within the discharge status variable. Records without explicit mortality labels were assigned programmatically to ensure consistency across the dataset. For clinical interpretability and model calibration, death was pre-specified as the positive outcome class in all analyses.

Only deaths confirmed during hospitalization were available for analysis; post-discharge mortality and long-term follow-up data were not captured in the administrative database. Each outcome corresponded to a unique hospitalization episode, verified through cross-referencing admission and discharge identifiers to prevent duplication.

### 2.3. Predictors

The five routinely collected variables were included for model development, representing demographic characteristics, disease burden, and care process indicators. Demographic predictors comprised age, treated as a continuous variable in years, and sex. Disease burden was captured using the HFRS and the Charlson Comorbidity Index (CCI), both derived from ICD-10 diagnostic codes and widely validated as measures of frailty and multimorbidity in hospitalized populations. Health service utilization was represented by length of hospital stay, expressed in days, which reflects clinical severity and complexity of care during admission.

Categorical variables were encoded using one-hot encoding, while continuous variables were standardized prior to model training to improve numerical stability and comparability across algorithms. All predictors were available at or early during hospitalization, allowing for potential application of the models for early risk stratification. Each row of the analytical dataset represented a unique hospitalization episode. The final modeling matrix consisted of five explanatory variables routinely available in administrative hospital records—age, sex, Hospital Frailty Risk Score, Charlson Comorbidity Index, and length of hospital stay—and one binary response variable indicating survival or in-hospital death.

### 2.4. Data Management and Preprocessing

To ensure robustness, consistency, and reproducibility across heterogeneous administrative exports, a standardized data management and preprocessing workflow was implemented prior to model development. Raw datasets were imported using an automated parser capable of recognizing multiple delimiters and character encodings commonly observed in nationwide CSV files. Column headers were normalized to lower-case ASCII format and converted to snake_case notation to ensure uniform variable naming. Synonym mapping was applied to harmonize equivalent labels across files and prevent inconsistencies during merging and analysis.

Variable types were systematically validated and recoded to ensure compatibility across algorithms. Continuous variables, including age, HFRS, CCI, and length of hospital stay, were converted to numeric format. Categorical predictors were transformed into binary or nominal representations as appropriate. Records lacking outcome information or containing no predictor data were excluded to preserve analytical validity.

Missing data were handled using simple and reproducible imputation strategies: the median was applied to continuous variables and the mode to categorical variables. This approach minimized distributional distortion while maintaining computational efficiency and interpretability in large administrative datasets. Numerical features were subsequently standardized using z-score normalization to reduce scale-related bias during optimization, whereas categorical variables were encoded using a OneHotEncoder configured to ignore previously unseen categories.

To prevent data leakage, all preprocessing steps—including imputation, scaling, and encoding—were encapsulated within scikit-learn Pipelines and fitted exclusively on the training subset. The same fitted transformations were then applied to the independent test data. This design ensured strict separation between training and evaluation phases, safeguarding the validity of performance estimates and enhancing methodological transparency.

### 2.5. Data Partition and Validation Strategy

To obtain an unbiased estimate of predictive performance and ensure generalizability, the dataset was randomly divided into independent training and testing subsets using an 80/20 stratified split. Stratification preserved the original distribution of the outcome classes (survival vs. in-hospital death) in both subsets, which was particularly relevant given the relatively lower prevalence of mortality events.

The final analytical dataset included 58,306 hospitalization episodes, each represented by five explanatory variables—age, sex, Hospital Frailty Risk Score, Charlson Comorbidity Index, and length of hospital stay—and one binary response variable indicating survival or in-hospital death. Based on the 80/20 stratified split, 46,645 episodes were assigned to the training subset and 11,661 episodes to the independent test subset. Prior to model development, all variables were inspected for completeness, consistency, and plausible value ranges. Continuous predictors were converted to numeric format, missing values were imputed using the median, and numerical features were standardized using z-score normalization within the preprocessing pipeline. Categorical variables were encoded using one-hot encoding. Extreme values were not removed automatically, as they may represent clinically plausible observations in administrative hospital data, particularly for age, frailty burden, comorbidity burden, and length of stay. Instead, only implausible or structurally inconsistent records were excluded during the data quality control process. Because in-hospital mortality was less frequent than survival, the outcome distribution was imbalanced; therefore, stratified splitting and algorithm-level class weighting were used to preserve class distribution and reduce bias toward the majority class.

Each hospitalization episode was assigned exclusively to one subset to maintain strict independence between training and evaluation data. No records were shared across partitions. Randomization was performed using a fixed seed (random_state = 42) to guarantee full reproducibility of the data split.

All model development procedures—including preprocessing transformations, hyperparameter tuning, and model fitting—were conducted exclusively on the training set. The independent test set was held out and used only once for final performance evaluation. This separation prevented information leakage and ensured that reported metrics reflected true out-of-sample predictive ability rather than optimistic in-sample estimates.

To further assess model robustness during development, stratified k-fold cross-validation was performed within the training subset. Cross-validation was restricted exclusively to the training data, whereas the independent test set remained untouched until the final evaluation. This strategy allowed the stability of model performance to be examined without introducing information leakage or compromising the unbiased nature of the final out-of-sample performance estimates.

This validation framework was selected to approximate real-world deployment conditions, where models trained on historical data are subsequently applied to unseen patient populations.

### 2.6. Model Development

A benchmark framework was adopted to systematically compare multiple supervised machine learning algorithms with different underlying mathematical assumptions, including linear, distance-based, tree-based, ensemble, and neural network approaches. This strategy aimed to identify the most robust and generalizable classifier rather than relying on a single modeling technique.

Fourteen classification algorithms were implemented: Extra Trees (Extremely Randomized Trees), Random Forest, Gradient Boosting, XGBoost, LightGBM, CatBoost, k-Nearest Neighbors (kNN), Multilayer Perceptron (MLP) neural network, Decision Tree, Support Vector Classifier (SVC), AdaBoost, Gaussian Naïve Bayes, Linear Discriminant Analysis (LDA), and Logistic Regression. These models were selected to represent a broad spectrum of complexity levels and learning paradigms commonly applied in clinical prediction tasks.

All algorithms were implemented within a standardized scikit-learn Pipeline architecture integrating preprocessing transformations and model fitting into a single workflow. This design minimized the risk of data leakage and ensured consistent handling of predictors across models. Hyperparameter optimization was performed within the training subset using GridSearchCV with stratified k-fold cross-validation. The search grids were defined according to commonly tuned parameters for each algorithm, including the number of estimators, maximum tree depth, learning rate, regularization terms, number of neighbors, kernel parameters, and neural network architecture, as applicable. The area under the receiver operating characteristic curve was used as the optimization criterion. The independent test set was not used during hyperparameter tuning and was reserved exclusively for final model evaluation. For stochastic algorithms, random seeds were fixed (random_state = 42) to guarantee deterministic outputs. The best hyperparameter configuration identified for each model was subsequently refitted on the complete training set before evaluation on the independent test set.

Model training was performed exclusively on the training subset. No information from the independent test set was used during fitting or tuning. Each model generated predicted probabilities for the outcome, which were subsequently used for discrimination analysis and clinical interpretability. This unified framework enabled direct and fair comparison of performance metrics across all classifiers under identical preprocessing and validation conditions.

To quantify the incremental contribution of key predictors to model performance, an ablation analysis was conducted using the best-performing algorithm. The Extra Trees classifier was retrained under different predictor configurations: the full model including age, sex, Hospital Frailty Risk Score, Charlson Comorbidity Index, and length of hospital stay; a model excluding HFRS; a model excluding length of hospital stay; a demographic-only model including age and sex; and a frailty-only model including HFRS alone. All ablation models were trained and evaluated using the same preprocessing pipeline, stratified data partition, and performance metrics as the primary analysis. The independent test set was used only for final evaluation. Differences in AUC-ROC across configurations were examined to assess the incremental predictive contribution of each predictor set.

### 2.7. Performance Metrics

Model performance was evaluated exclusively on the independent test set to obtain an unbiased estimate of predictive accuracy and generalizability. No observations from the test subset were used during model training or preprocessing, ensuring strict out-of-sample evaluation.

Discriminative ability was primarily quantified using the area under the receiver operating characteristic curve (AUC-ROC), which measures the capacity of the model to distinguish between survivors and non-survivors across all possible classification thresholds. The AUC-ROC was selected as the main metric because of its robustness to class imbalance and its widespread use in clinical prediction research.

To provide a comprehensive assessment of classification behavior, additional performance indicators were calculated, including accuracy, precision (positive predictive value), recall (sensitivity), F1-score, and the Matthews Correlation Coefficient (MCC). Given the imbalanced outcome distribution, MCC was computed as a complementary metric because it incorporates true positives, true negatives, false positives, and false negatives into a single balanced measure of binary classification performance.

For each classifier, predicted probabilities were used to construct ROC curves. Individual curves were exported in high-resolution format (600 dpi), and a combined summary figure was generated to facilitate visual comparison across models. When an algorithm produced decision scores rather than probabilities, a sigmoid transformation was applied to enable probability-based discrimination analysis without altering relative ranking or AUC values.

All metrics were computed under identical evaluation conditions to ensure fair and reproducible comparison among models.

### 2.8. Model Explainability

To enhance clinical interpretability and facilitate translation of model predictions into actionable insights, post hoc explainability analyses were conducted using SHapley Additive exPlanations (SHAP). This framework quantifies the contribution of each predictor to individual predictions based on cooperative game theory principles, allowing decomposition of the model output into additive feature effects.

Interpretability analyses were performed for the best-performing algorithm according to AUC-ROC. The Extra Trees classifier demonstrated the highest overall discriminative performance in the benchmark comparison and was therefore selected for detailed explanation. Given its tree-based architecture, SHAP values were computed using the TreeExplainer method, which provides exact and computationally efficient attribution estimates for ensemble decision tree models.

Both global and local interpretability outputs were generated. Global explanations included bar plots of mean absolute SHAP values to rank overall predictor importance, as well as beeswarm plots illustrating the distribution and directional effect of each feature across the population. Local explanations were assessed through individual waterfall plots and heatmaps to visualize how specific predictors contributed to the predicted risk for each patient. Additionally, dependence plots were constructed for the most influential variables to explore potential nonlinear relationships and interactions between predictors.

SHAP values were expressed in log-odds space, where positive values indicated increased predicted probability of in-hospital mortality and negative values reflected protective effects. All visualizations were exported at high resolution (600 dpi) to ensure publication quality and reproducibility.

### 2.9. SHAP-Based Risk Equation

Finally, to provide an explicit quantitative representation of individualized risk, an additive SHAP-based risk equation was derived for the best-performing model according to AUC-ROC. Because SHAP values are additive in log-odds space for tree-based algorithms, each patient’s prediction can be decomposed into the sum of a baseline model output and the individual contributions of all predictors. Formally, the model output is expressed as:

(1) $$logit\left(p\right)=\beta_{0}+\sum_{i}\phi_{i}$$

(2) $$p=\frac{1}{1+exp\left(-logit\left(p\right)\right)}$$

(3) $$RR=\frac{p}{p_{0}}$$

where *β*_0_ represents the SHAP base value, corresponding to the expected model output across the entire population, *ϕᵢ* denotes the contribution of predictor *i* to the individual prediction, *p* is the predicted probability of in-hospital mortality obtained through the logistic transformation, and *RR* represents the relative risk compared with the baseline probability $p_{0}$.

This formulation enables direct decomposition of each patient’s predicted risk into additive feature contributions, providing transparent, quantitative, and patient-specific explanations of the model’s predictions. Consequently, the contribution of each clinical variable can be interpreted as an increase or decrease in the log-odds of mortality, thereby facilitating clinical interpretability and supporting individualized risk assessment.

### 2.10. Handling of Class Imbalance

Given the relatively lower prevalence of in-hospital mortality compared with survival, the dataset exhibited moderate class imbalance. To mitigate potential bias toward the majority class and improve sensitivity for high-risk patients, imbalance was addressed through algorithm-level weighting strategies rather than synthetic resampling.

Specifically, the parameter class_weight = “balanced” was applied to all classifiers supporting this option, automatically adjusting class contributions in inverse proportion to their observed frequencies during model training. This approach increased the penalization of misclassified deaths while preserving the original empirical distribution of the data.

In addition, the stratified train–test split maintained consistent outcome proportions across both subsets, ensuring comparable class representation during training and evaluation. No oversampling, undersampling, or synthetic data generation techniques—such as SMOTE or related methods—were implemented in the primary analysis, as these approaches may artificially distort the underlying clinical distribution and reduce external validity in administrative healthcare datasets. Because no synthetic resampling procedures were applied, MCC was additionally considered to complement the conventional classification metrics under class imbalance.

This strategy aimed to balance predictive performance with real-world generalizability, prioritizing clinically meaningful detection of mortality events while preserving the natural characteristics of the population.

### 2.11. Statistical Considerations

The unit of analysis was the hospitalization episode, with each record corresponding to a distinct patient admission. Exclusions were limited to essential variables required for cohort definition or outcome ascertainment, including age, sex, discharge status, or absence of all predictor data, in order to preserve the representativeness of the nationwide administrative cohort. Missing values in predictor variables were addressed using median imputation for continuous features and mode imputation for categorical features within the preprocessing pipeline, as previously described.

Descriptive statistics were used to summarize the demographic and clinical characteristics of the study population. Continuous variables were reported as mean ± standard deviation, and categorical variables as frequencies and percentages. To explore baseline differences between survivors and non-survivors, non-parametric comparisons were performed using the Kruskal–Wallis test for continuous variables, given the expected non-normal distribution of administrative data. A significance level of *p* < 0.05 was adopted for these exploratory comparisons.

Because the primary objective of the study was predictive modeling rather than causal inference, these statistical analyses were intended solely for cohort characterization and were conducted independently of model training. No hypothesis-driven feature selection or inferential modeling influenced the machine learning workflow. Model comparisons were therefore based exclusively on out-of-sample predictive metrics.

Hospital-level clustering effects were not explicitly modeled, as the analytical focus was placed on patient-level mortality risk prediction and maximizing generalizability across the national public hospital network. To ensure full reproducibility, the random seed parameter (random_state = 42) was fixed across all stages of the workflow, including data partitioning, model training, and evaluation. Confidence intervals for the AUC were not calculated in the primary analysis to prioritize computational efficiency; however, these can be readily obtained using stratified bootstrap resampling if required.

### 2.12. Software and Reproducibility

All analyses were conducted using Python 3.11 within a Jupyter Notebook 7.5 environment [31]. The computational workflow was implemented using widely adopted open-source libraries, including pandas for data manipulation, NumPy for numerical operations, scikit-learn for preprocessing pipelines and machine learning algorithms, matplotlib for visualization, and SHAP for model interpretability [32]. The analytical pipeline was designed to ensure full reproducibility and transparency. All preprocessing, model training, and evaluation steps were executed programmatically using standardized scripts, thereby minimizing manual intervention and reducing the risk of procedural inconsistencies. Performance metrics and graphical outputs—including ROC curves and SHAP visualizations—were automatically generated and exported in publication-quality resolution.

Random seeds were fixed across all stochastic procedures to guarantee deterministic results. The complete workflow can be readily replicated by re-running the scripts on the original administrative datasets. When required, the environment can be containerized (e.g., using Docker 29.0) to facilitate portability across computational platforms and long-term reproducibility.

## 3. Results

Table 1 summarizes baseline characteristics stratified by sex and survival status. Among men, non-survivors were older (78.6 ± 9.3 vs. 76.4 ± 9.4 years), had longer hospital stays (10.5 ± 17.2 vs. 9.0 ± 12.8 days), higher frailty burden as measured by HFRS (7.6 ± 5.4 vs. 4.8 ± 4.8), and a higher CCI (2.4 ± 2.0 vs. 2.1 ± 1.8) compared with survivors (all *p* < 0.001). Similarly, among women, non-survivors were older (80.9 ± 9.6 vs. 78.3 ± 9.8 years), had longer hospital stays (8.8 ± 14.5 vs. 8.1 ± 10.0 days), higher HFRS (7.9 ± 5.5 vs. 5.1 ± 4.9), and higher CCI (2.3 ± 1.9 vs. 2.0 ± 1.7) than survivors (all *p* < 0.001). Overall, the sex-stratified pattern indicates that older age, greater frailty (HFRS), higher comorbidity burden (Charlson Comorbidity Index), and longer hospitalization were consistently associated with in-hospital death across both men and women.

Fourteen supervised machine learning algorithms were trained and evaluated using an independent stratified test set to compare their ability to predict in-hospital mortality. Overall discriminative performance varied across models, with ensemble tree-based approaches consistently outperforming linear and distance-based classifiers. The Extra Trees classifier demonstrated the highest discrimination, achieving the largest AUC-ROC, followed closely by other gradient boosting and ensemble methods. In contrast, simpler probabilistic and linear models showed comparatively lower sensitivity for mortality detection. A complete summary of accuracy, precision, recall, F1-score, and AUC-ROC for all algorithms is presented in Table 2.

Given the imbalanced outcome distribution, MCC was also examined as a complementary metric. The Extra Trees classifier achieved an MCC of 0.56, supporting its balanced classification performance under class imbalance, consistent with its superior AUC-ROC, F1-score, and recall.

Ablation analysis of the Extra Trees classifier was performed to examine the incremental contribution of key predictors to mortality prediction. The full model, including age, sex, HFRS, CCI, and length of hospital stay, achieved the highest discriminative performance, with an AUC-ROC of 0.862. When HFRS was removed from the model, the AUC-ROC decreased to 0.792, supporting the incremental predictive value of frailty beyond age, sex, comorbidity burden, and length of stay. Excluding length of hospital stay reduced the AUC-ROC to 0.834, suggesting that this variable also contributed additional prognostic information. The demographic-only model, including age and sex, showed lower discrimination, with an AUC-ROC of 0.641, whereas the frailty-only model retained moderate predictive capacity, with an AUC-ROC of 0.774. These findings were consistent with the SHAP analysis, which identified HFRS as the strongest contributor to model predictions.

**Figure 1:**
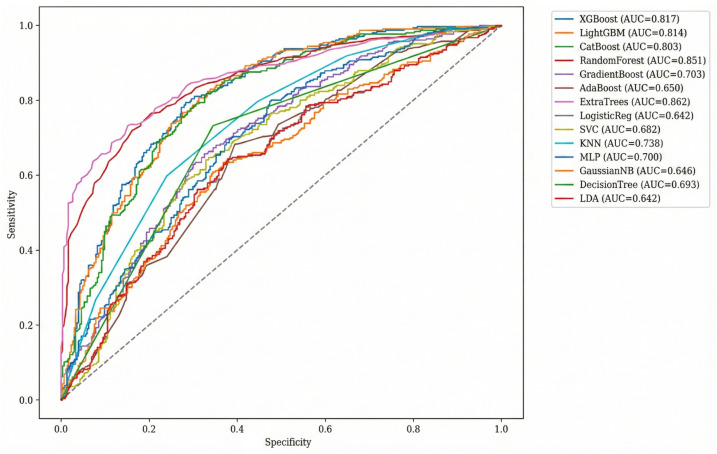
Receiver operating characteristic (ROC) curves for all supervised machine learning models evaluated on the independent test set. Receiver operating characteristic curves illustrate the discriminative performance of the 14 supervised classifiers for predicting in-hospital mortality. Curves closer to the upper-left corner indicate superior discrimination. Ensemble tree-based methods demonstrated consistently higher performance, with the Extra Trees classifier achieving the largest area under the curve (AUC-ROC), followed by Random Forest and gradient boosting approaches. The diagonal dashed line represents the reference line for chance-level discrimination, corresponding to an AUC of 0.50; curves above this line indicate performance better than random classification.

Receiver operating characteristic analysis further confirmed differences in discriminative ability among classifiers. Ensemble tree-based models consistently achieved superior separation between survivors and non-survivors across all probability thresholds. The Extra Trees classifier demonstrated the highest discriminative performance, followed by Random Forest and gradient boosting approaches. In contrast, linear and probabilistic classifiers showed reduced curve separation and lower overall discrimination. Visual comparison of ROC curves across all models is presented in Figure 1.

**Figure 2:**
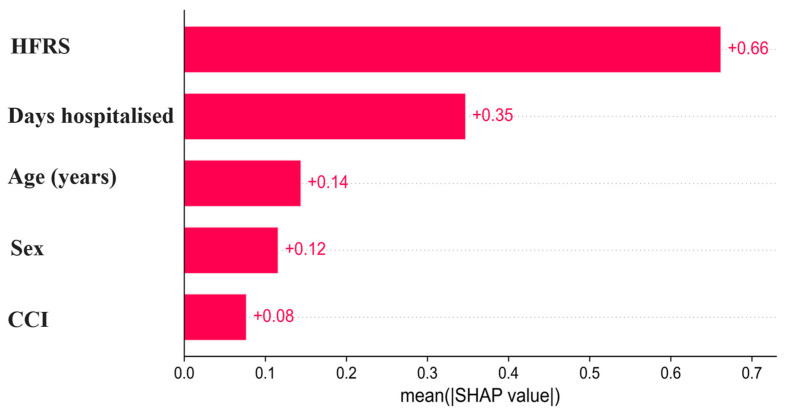
Global feature importance based on mean absolute SHAP values for the Extra Trees mortality prediction model. Mean absolute SHAP values (mean |SHAP|) quantify the average magnitude of each predictor’s contribution to the model output across all patients, independent of direction. Higher values indicate greater overall influence on mortality predictions. The Hospital Frailty Risk Score (HFRS) showed the largest contribution (0.66), followed by length of hospital stay (0.35). Age (0.14), sex (0.12), and Charlson Comorbidity Index (CCI) (0.08) demonstrated smaller but still meaningful contributions. This ranking reflects the relative importance of predictors at the population level.

Figure 2 displays the global feature importance ranking derived from the mean absolute SHAP values of the Extra Trees classifier. The Hospital Frailty Risk Score (HFRS) showed the largest contribution to model predictions (mean |SHAP| = 0.66), followed by length of hospital stay (0.35). Age (0.14), sex (0.12), and CCI (0.08) demonstrated smaller but still relevant contributions. These results indicate that frailty burden and healthcare complexity were the dominant drivers of predicted mortality risk at the population level.

**Figure 3:**
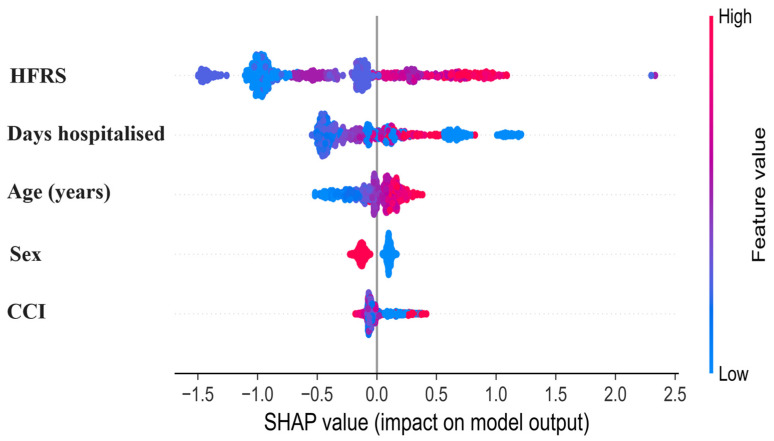
SHAP summary (beeswarm) plot illustrating the distribution and direction of individual feature contributions to predicted in-hospital mortality. Each dot corresponds to one hospitalization episode. The x-axis shows SHAP values in log-odds space, where positive values indicate increased mortality risk and negative values indicate decreased risk. Colors represent original feature magnitude (red = high, blue = low). Features are ordered by global importance.

To further characterize the direction and variability of feature effects, SHAP summary (beeswarm) plots were generated for the Extra Trees classifier (Figure 3). Each point represents an individual hospitalization, with the horizontal position indicating the SHAP value (log-odds contribution to predicted mortality) and color representing the original feature value (red = higher values; blue = lower values).

Higher frailty burden (HFRS) showed the widest dispersion and predominantly positive SHAP values, indicating that greater frailty substantially increased predicted mortality risk. Longer hospital stays and older age demonstrated similar directional patterns, with higher values shifting predictions toward increased risk and lower values toward reduced risk.

Sex displayed a bimodal distribution consistent with its binary encoding, reflecting differential risk contributions between categories rather than a continuous gradient. The CCI exhibited smaller but consistently positive contributions at higher values, suggesting a modest incremental effect on mortality prediction.

**Figure 4:**
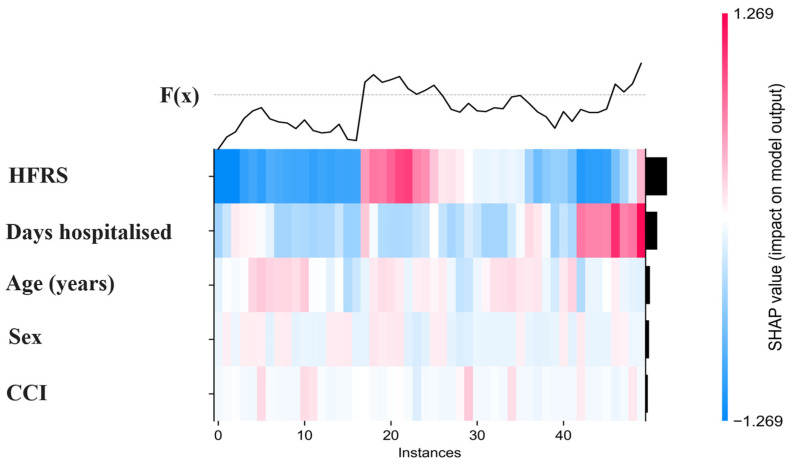
SHAP heatmap showing individual feature contributions for 50 representative hospitalization episodes. Each column corresponds to one hospitalization episode, and each row corresponds to one predictor. Colors represent SHAP values on the log-odds scale, where red indicates a positive contribution to the predicted risk of in-hospital mortality and blue indicates a negative contribution. The upper black line, F(x), represents the model output for each individual episode, reflecting the predicted log-odds across instances. The black bars on the right summarize the overall magnitude of each predictor’s contribution within the displayed sample. The plot illustrates inter-individual variability in how predictors combine to determine personalized risk estimates.

To visualize individual-level contribution patterns across multiple observations simultaneously, a SHAP heatmap was generated for a representative subset of 50 hospitalization episodes (Figure 4). This visualization displays the SHAP values of each predictor for each patient, where colors indicate the magnitude and direction of contributions to the model output (red = positive contribution to predicted mortality risk; blue = negative contribution). The heatmap highlights heterogeneity in how predictors influence individual risk estimates. Consistent with previous analyses, the Hospital Frailty Risk Score (HFRS) and length of hospital stay showed the largest and most variable contributions across patients, whereas age, sex, and comorbidity index exhibited smaller and more homogeneous effects. These patterns illustrate how the model integrates multiple features additively to generate personalized mortality predictions.

**Figure 5:**
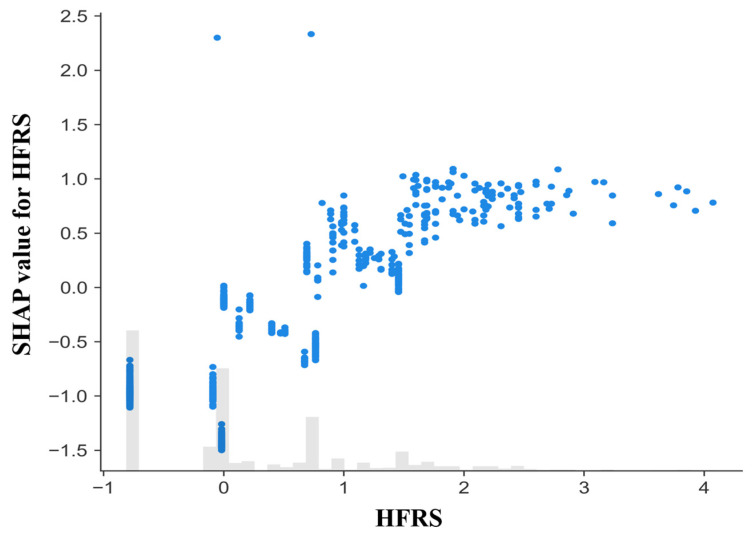
SHAP dependence plot for the Hospital Frailty Risk Score (HFRS). Each point represents one hospitalization episode. The x-axis shows the original HFRS value and the y-axis shows its SHAP contribution to the model output (log-odds scale). Positive values indicate increased predicted mortality risk and negative values indicate reduced risk. The histogram at the bottom represents the distribution of HFRS values in the cohort.

To explore the functional relationship between frailty and its contribution to model predictions, a SHAP dependence plot was generated for the Hospital Frailty Risk Score (HFRS) (Figure 5). The x-axis represents the original HFRS value, while the y-axis displays the corresponding SHAP value, reflecting the feature’s contribution to the predicted mortality risk on the log-odds scale. A clear monotonic pattern was observed: lower frailty scores were generally associated with negative SHAP values, indicating reduced predicted mortality risk, whereas higher scores corresponded to progressively positive contributions. The magnitude of SHAP values increased with frailty severity, suggesting that patients with greater frailty exerted stronger influence on model output. The distribution histogram indicates that most observations clustered at lower HFRS values, with fewer but more influential contributions observed among highly frail individuals. These findings are consistent with the global and summary analyses, confirming frailty as the most influential predictor in the model.

**Figure 6:**
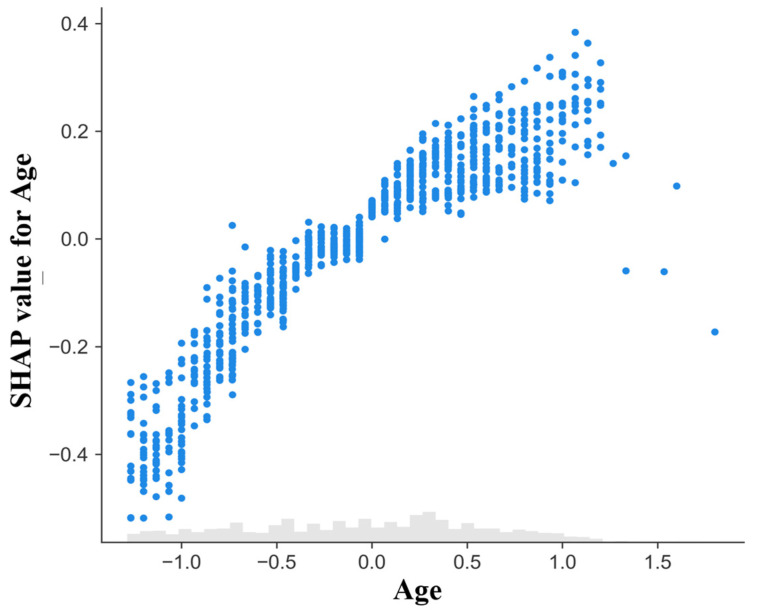
SHAP dependence plot for age. Each point represents one hospitalization episode. The x-axis shows standardized age and the y-axis shows its SHAP contribution to the model output (log-odds scale). Positive values indicate increased predicted mortality risk and negative values indicate reduced risk. Vertical dispersion reflects inter-individual variability in contributions. The gray histogram at the bottom represents the marginal distribution of standardized age values in the analyzed sample.

A SHAP dependence plot was constructed to examine the relationship between age and its contribution to mortality predictions (Figure 6). The x-axis displays standardized age values, while the y-axis represents the corresponding SHAP value, indicating the feature’s contribution to the model output on the log-odds scale.

An overall monotonic trend was observed, with younger ages generally associated with negative SHAP values (lower predicted mortality contribution) and progressively older ages corresponding to increasingly positive contributions. The magnitude of SHAP values increased gradually across the age range, suggesting that age exerted a cumulative influence on model predictions.

Vertical dispersion at similar age levels indicates heterogeneity in individual contributions, reflecting interactions with other patient characteristics. Overall, age demonstrated a consistent but moderate effect compared with frailty and length of stay.

**Figure 7:**
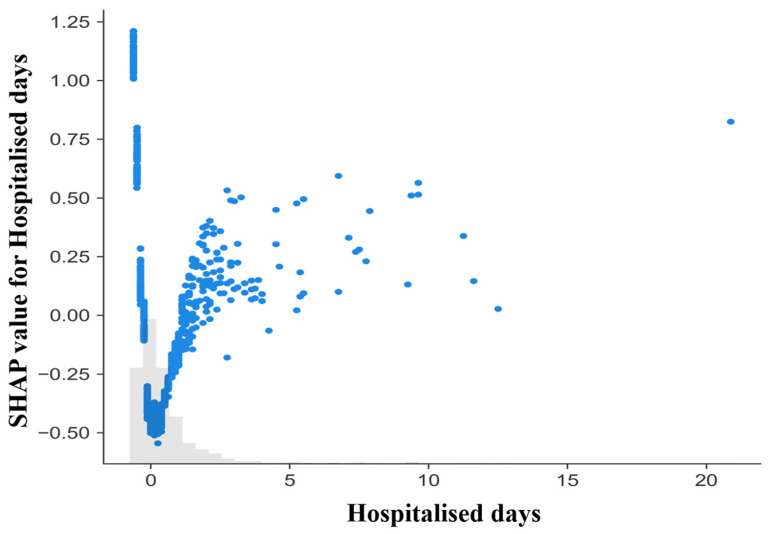
SHAP dependence plot for length of hospital stay. Each point represents one hospitalization episode. The x-axis shows days hospitalized and the y-axis shows the SHAP contribution to the model output (log-odds scale). Positive values indicate increased predicted mortality risk and negative values indicate reduced risk. Vertical dispersion reflects inter-individual variability in contributions. Values of 0 days reflect same-day hospitalization episodes based on administrative admission and discharge dates, rather than absence of hospital care The gray histogram at the bottom represents the marginal distribution of hospitalization days in the analyzed sample.

A SHAP dependence plot was generated to assess the relationship between length of hospital stay and its contribution to model predictions (Figure 7). The x-axis represents the number of hospitalization days, while the y-axis displays the corresponding SHAP value, reflecting the feature’s contribution to predicted mortality on the log-odds scale. An overall upward and non-linear pattern was observed. Shorter hospital stays were generally associated with negative or near-zero SHAP values, indicating smaller contributions to predicted mortality risk, whereas longer stays produced progressively positive contributions. The magnitude of SHAP values increased with length of stay, suggesting that prolonged hospitalization exerted a stronger influence on model output. Considerable vertical dispersion was present across the range of stay durations, indicating heterogeneity in how this feature interacted with other predictors at the individual level. Overall, length of stay emerged as one of the most influential contributors after frailty.

**Figure 8:**
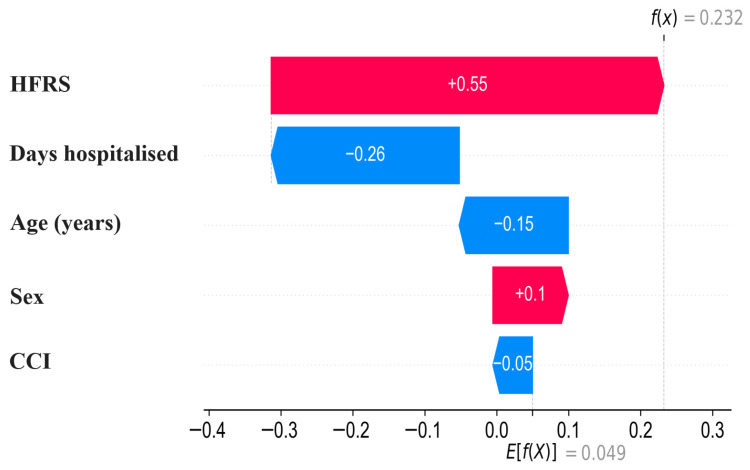
SHAP waterfall plot illustrating the additive contributions of individual predictors to a single patient’s mortality prediction. The prediction starts from the model baseline value (E[f(X)] = 0.049), corresponding to the average model output across the population. Each subsequent bar represents the SHAP value of an individual predictor, expressed on the log-odds scale. Red bars indicate predictors that increase the model output and contribute positively to the predicted mortality risk, whereas blue bars indicate predictors that decrease the model output and contribute negatively to the predicted mortality risk. In this example, the Hospital Frailty Risk Score contributes +0.55 and age contributes +0.10, whereas length of stay (−0.26) and sex (−0.15) reduce the cumulative contribution. The sex category for the illustrated patient was [male/female], indicating that this category decreased the predicted mortality contribution for this specific case.

To illustrate how individual predictions are constructed, a SHAP waterfall plot was generated for a representative hospitalization episode (Figure 8). The prediction begins at the model baseline value, corresponding to the expected output across the population, and is subsequently adjusted by the additive contributions of each predictor. Positive SHAP values shift the prediction toward higher mortality probability, whereas negative values reduce the predicted contribution on the log-odds scale. In this example, the HFRS produced the largest positive contribution, followed by age, whereas length of stay and sex generated negative contributions. Specifically, the sex category of the illustrated patient was [male/female], and in this individual prediction this category reduced the predicted mortality contribution. The cumulative sum of these feature effects resulted in the final predicted probability for this individual. This visualization demonstrates how the model integrates multiple predictors additively to generate patient-specific risk estimates.

## 4. Discussion

In this nationwide retrospective cohort of 58,306 hospitalization episodes of older adults admitted with CAP in Chile, we developed and internally validated a benchmark set of supervised machine learning models to predict in-hospital mortality using five routinely available predictors (age, sex, HFRS, CCI, and length of stay). Overall, ensemble tree-based approaches achieved the best discrimination, with the Extra Trees classifier showing the highest AUC-ROC (0.862) and the most balanced performance across complementary metrics. Explainability analyses based on SHAP consistently identified frailty burden (HFRS) as the dominant contributor to mortality predictions, followed by length of hospital stay and age, whereas comorbidity burden (CCI) and sex exhibited smaller effects. Collectively, these findings support the feasibility of leveraging administrative risk scores and basic clinical descriptors to enable early mortality risk stratification in older patients hospitalized with pneumonia within the Chilean public hospital network.

When compared with recent studies applying machine learning to pneumonia prognosis, our findings are consistent with evidence showing that non-linear and ensemble-based models can improve mortality prediction over traditional regression approaches. Cillóniz et al. [20] demonstrated the usefulness of machine learning for mortality prediction in patients with community-acquired pneumonia, while Jeon et al. [22] and Pan et al. [13] reported robust performance of machine learning models in ICU and severe pneumonia cohorts. In addition, Li et al. [28] highlighted the value of SHAP-based explainability for interpreting mortality prediction models in patients with pneumonia. Unlike many previous studies, which often relied on ICU populations, clinical severity variables, laboratory parameters, or single-center datasets, the present study used a nationwide administrative database and a parsimonious set of routinely available predictors. Therefore, our findings extend previous evidence by showing that good discrimination can be achieved using administrative hospital data, particularly when frailty is incorporated through the Hospital Frailty Risk Score.

Building on these findings, the superior performance of ensemble tree-based algorithms over linear and probabilistic classifiers likely reflects their ability to model complex, non-linear relationships and higher-order interactions between clinical vulnerability indicators. Administrative healthcare data rarely follow simple parametric structures, and predictors such as frailty, multimorbidity, and healthcare utilization often interact synergistically rather than additively. Tree ensembles are particularly well suited to capture these patterns without imposing distributional assumptions, which may explain their consistent advantage in discrimination [33]. The observed performance gradient—from logistic regression and LDA to gradient boosting and Extra Trees—suggests that increasing model flexibility provides meaningful gains in predictive accuracy when risk emerges from heterogeneous clinical trajectories.

The predominance of frailty burden (HFRS) as the leading predictor is clinically coherent and reinforces the central role of biological vulnerability in determining outcomes among older inpatients with pneumonia [6,34,35]. Frailty captures multidimensional physiological reserve, cumulative deficits, and reduced capacity to respond to acute stressors, factors that may not be fully represented by chronological age or individual disease diagnoses alone [10]. From a pathophysiological perspective, frail individuals often exhibit impaired immune response, sarcopenia, diminished cardiorespiratory reserve, and increased susceptibility to complications such as delirium or deconditioning, all of which may contribute to poorer in-hospital outcomes [4,36,37]. Consequently, it is plausible that a frailty-oriented index provides a more integrative representation of short-term mortality risk than traditional comorbidity counts.

Length of hospital stay emerged as the second most influential contributor to model predictions. Although this variable should not be interpreted causally, it likely acts as a proxy for clinical severity, treatment complexity, and early complications occurring during admission [38,39]. Prolonged stays may reflect greater physiological instability, more comorbidities, need for intensive monitoring, or slower recovery trajectories, which collectively signal higher mortality risk [38,39]. Importantly, extended hospitalization is frequently accompanied by prolonged immobilization, which has been associated with accelerated muscle mass and strength loss, functional decline, increased risk of complications such as thromboembolism and infections, and overall physiological deconditioning [40,41,42,43]. These processes may further exacerbate vulnerability and contribute to poorer outcomes. Conversely, shorter stays were generally associated with lower predicted risk. Taken together, these findings highlight how healthcare process indicators embedded in administrative records can carry substantial prognostic information, complementing baseline patient characteristics.

Age showed a consistent but more gradual effect on predicted mortality, suggesting a cumulative rather than threshold-based contribution. This pattern is compatible with the well-established association between aging and progressive decline in functional reserve, immune competence, and resilience to acute infections [44,45]. In contrast, the CCI demonstrated comparatively smaller contributions once frailty and age were considered. This may indicate partial redundancy between multimorbidity and frailty constructs, or that comorbidity burden alone does not fully capture the functional and physiological vulnerability that determines short-term inpatient prognosis [6]. Similarly, sex exhibited limited incremental predictive value after accounting for other risk factors, suggesting that sex-related differences may be mediated through broader health status indicators rather than acting as independent drivers of mortality risk in this context [44,46].

From a translational standpoint, the ability to achieve good discrimination using only five routinely available predictors has important practical implications. Because these variables are readily extractable from standardized administrative records, the proposed framework could be implemented at scale without requiring additional laboratory tests, imaging, or manual data collection. This feature is particularly relevant for public healthcare systems with constrained resources, where simple, automated tools for early risk stratification may help prioritize monitoring, guide multidisciplinary interventions, or inform discharge planning. Nevertheless, clinical integration should proceed cautiously, emphasizing calibration, prospective validation, and evaluation of decision impact before operational deployment.

From a clinical perspective, these findings have several practical implications. Because all predictors are routinely available administrative or early hospitalization variables, the proposed models could be implemented automatically within hospital information systems without requiring additional tests or data collection [47,48]. This feasibility enables near-real-time risk stratification at or shortly after admission, supporting early identification of high-risk older adults who may benefit from closer monitoring, proactive geriatric assessment, escalation of care, or prioritization for intermediate or intensive care resources [20]. Conversely, low-risk patients could be considered for standard ward management or earlier discharge planning, potentially optimizing bed utilization. Importantly, the explainability framework based on SHAP provides transparent patient-level risk decomposition, allowing clinicians to understand which factors—such as frailty or prolonged hospitalization—drive the prediction and facilitating trust and clinical adoption [49]. Therefore, this approach may serve not only as a predictive tool but also as a decision-support aid to guide individualized management in resource-constrained public healthcare settings. Beyond immediate clinical application, these findings also have implications for future research and digital health implementation. By showing that frailty-informed, explainable machine learning models can achieve good discrimination using routinely collected administrative data, this study supports the development of scalable prediction tools that may be adapted to other high-burden conditions in older adults. In addition, the use of a nationwide public database provides a relevant framework for evaluating how such models could be embedded into hospital information systems, monitored over time, and prospectively recalibrated to maintain performance across changing patient profiles and healthcare contexts.

This study has several limitations that should be considered when interpreting the findings. First, the retrospective design based on administrative discharge data may be subject to information bias and coding inaccuracies. Although ICD-10 coding is standardized and routinely audited within the Chilean public health system, misclassification of diagnoses, comorbidities, or outcomes cannot be fully excluded. Additionally, administrative registries lack clinical granularity, and important physiological parameters—such as vital signs, laboratory biomarkers, imaging findings, and early severity scores—were unavailable. The absence of these variables may have limited model precision and introduced residual confounding, as risk estimation relied primarily on demographic characteristics, comorbidity burden, frailty indices, and healthcare utilization proxies. Second, mortality was defined exclusively as in-hospital death recorded at discharge. Consequently, deaths occurring shortly after discharge were not captured, precluding time-to-event analyses or competing-risk modeling. This restriction may have led to underestimation of short-term mortality risk and limits interpretation to the inpatient period only. Third, although the cohort represents the entire Chilean public hospital network and therefore provides strong internal representativeness, external validation beyond this setting was not performed. Differences in healthcare organization, admission thresholds, coding practices, or case-mix across countries or private healthcare systems may affect transportability. Prospective, temporal, and geographic validation studies are necessary before broader clinical implementation. Fourth, some predictors—particularly length of hospital stay—may partially reflect events occurring during hospitalization rather than purely baseline risk. While such variables improve predictive performance, they should not be interpreted causally and may limit applicability for very early bedside decision-making unless models are recalibrated at predefined time points. Fifth, as with any machine learning approach, there is potential for overfitting and performance degradation over time due to changes in clinical practice, population characteristics, or coding standards (dataset shift). Although strict train–test separation and reproducible pipelines were used to mitigate optimistic bias, ongoing monitoring and periodic recalibration would be required for real-world deployment. Finally, SHAP-based analyses provide explanations of model behavior rather than causal inference. Feature contributions quantify how variables influence predictions within the trained algorithm but do not establish mechanistic or causal relationships. Therefore, interpretability results should be understood within a predictive—not etiological—framework.

Despite these limitations, this study presents notable strengths. The large nationwide cohort enhances representativeness and statistical power, the benchmark comparison across diverse algorithms provides a rigorous evaluation of modeling strategies, and the integration of explainability techniques offers transparency regarding how predictions are constructed. Together, these elements support both methodological robustness and clinical interpretability. Future research should prioritize temporal and geographic external validation, formal assessment of calibration and clinical utility (e.g., decision-curve analysis), and exploration of additional predictors when available, such as functional status or early physiological parameters. Prospective implementation studies will be essential to determine whether incorporating such models into routine care can improve outcomes or resource allocation in older adults hospitalized with pneumonia.

## 5. Conclusions

In this nationwide cohort, ensemble tree-based models incorporating administrative frailty measures demonstrated higher discriminative performance than traditional linear approaches under internal validation. Frailty emerged as the primary contributor to predicted mortality risk, supporting the potential utility of scalable risk stratification using routinely available hospital data. The integration of SHAP-based explainability provided transparent and clinically interpretable decomposition of individual predictions, enabling identification of patient-specific risk drivers and supporting trust in model outputs. Because all predictors are readily available during routine care, this approach may be feasibly implemented within hospital information systems to support early risk stratification and resource allocation in real-world public healthcare settings, although its impact on clinical outcomes requires validation in prospective trials. Future research should prioritize external validation across different hospitals and time periods, as well as prospective evaluation of the clinical impact of model-assisted decision-making.

## Figures and Tables

**Table 1:** Baseline demographic and clinical characteristics of the study population stratified by survival status and sex.

Sex	Variable	Alive (Mean ± SD)	Dead (Mean ± SD)	*p*-Value
Men	Age (years)	76.4 ± 9.4	78.6 ± 9.3	<0.001
	Days hospitalized	9.0 ± 12.8	10.5 ± 17.2	<0.001
	HFRS	4.8 ± 4.8	7.6 ± 5.4	<0.001
	CCI	2.1 ± 1.8	2.4 ± 2.0	<0.001
Women	Age (years)	78.3 ± 9.8	80.9 ± 9.6	<0.001
	Days hospitalized	8.1 ± 10.0	8.8 ± 14.5	<0.001
	HFRS	5.1 ± 4.9	7.9 ± 5.5	<0.001
	CCI	2.0 ± 1.7	2.3 ± 1.9	<0.001

CCI: Charlson Comorbidity Index; HFRS: Hospital Frailty Risk Score.

**Table 2:** Comparative Evaluation of Predictive Efficacy in Supervised Learning Algorithms.

Algorithm	AUC ROC	F1-Score	Recall	Accuracy	Precision
ExtraTrees	0.862	0.781	0.791	0.777	0.771
Random Forest	0.851	0.777	0.814	0.766	0.743
XGBoost	0.817	0.764	0.794	0.754	0.736
LightGBM	0.814	0.756	0.794	0.743	0.721
CatBoost	0.803	0.760	0.810	0.743	0.715
KNN	0.738	0.711	0.797	0.675	0.642
Gradient Boost	0.703	0.677	0.725	0.652	0.634
MLP	0.700	0.673	0.703	0.657	0.646
Decision Tree	0.693	0.706	0.732	0.693	0.681
SVC	0.682	0.666	0.709	0.643	0.627
AdaBoost	0.650	0.608	0.595	0.615	0.621
Gaussian NB	0.646	0.632	0.634	0.630	0.630
LDA	0.642	0.628	0.654	0.611	0.604
Logistic Reg	0.642	0.627	0.650	0.611	0.605

AUC-ROC, area under the receiver operating characteristic curve; F1-score, harmonic mean of precision and recall; Recall, sensitivity; Accuracy, overall classification accuracy; Precision, positive predictive value; ExtraTrees, Extremely Randomized Trees; Random Forest, random forest classifier; XGBoost, Extreme Gradient Boosting; LightGBM, Light Gradient Boosting Machine; CatBoost, Categorical Boosting; KNN, k-nearest neighbors; Gradient Boost, gradient boosting classifier; MLP, multilayer perceptron; SVC, support vector classifier; AdaBoost, adaptive boosting; Gaussian NB, Gaussian naïve Bayes; LDA, linear discriminant analysis; Logistic Reg, logistic regression.

## Data Availability

The data supporting the findings of this study are publicly available from the Open Data Portal of the National Health Fund (FONASA), Chile, at: https://datosabiertos.fonasa.cl/ (accessed on 12 September 2025).

## Abbreviations

The following abbreviations are used in this manuscript:

AUC-ROC	Area Under the Receiver Operating Characteristic Curve
CAP	Community-Acquired Pneumonia
CCI	Charlson Comorbidity Index
CSV	Comma-Separated Values
GBD	Global Burden of Disease
HFRS	Hospital Frailty Risk Score
ICD-10	International Classification of Diseases, Tenth Revision
kNN	k-Nearest Neighbors
LDA	Linear Discriminant Analysis
MLP	Multilayer Perceptron
SHAP	SHapley Additive exPlanations
SVC	Support Vector Classifier
